# Book Reviews

**Published:** 1915-12

**Authors:** 


### BOOK REVIEWS

---

**Books mentioned may be inspected at and ordered through this office. So far as possible, books received in any month will be reviewed in the issue of the second month following.**

---

**La Localisation des Corps Etrangers avec le Repereur Marion-danion.** Dr. Marion, Paris. This is an illustrated pamphlet.

In brief, the method consists in radiography from two stations accurately located by a holding apparatus. A compass-like apparatus, so to speak, sights the foreign body from the same stations and is then applied to the surface of the patient so as to point the way for exploration.

---

**Diseases of the Skin and the Eruptive Fevers.** By Jay Frank Schamberg, M. D., Professor of Dermatology and Infectious Diseases in the Philadelphia Polyclinic and College for Graduates in Medicine. Third edition, revised. Octavo of 585 pages, 248 illustrations. Philadelphia and London: W. B. Saunders Company, 1915. Cloth, $3.00 net.

Having reviewed previous editions, it is only necssary to say that this work is standard and of the highest order of merit, and that it has been brought down to date. It is a work intermediate between brief compends and large atlases, in other words, of the scope best adapted to practical use.

---

**Emergency Surgery.** John W. Sluss, A. M., M. D., Indianapolis. Published by P. Blakiston's Son & Co., Philadelphia. Limp leather, 832 pages, 685 illustrations, some in color. $4.00.

This is a systematically arranged, compact book, uniform with others of the series. The third edition is so thoroughly up to date as to include the experience of the current year in the European War, with illustrations. It is a logical part of the surgeon's equipment for preparedness in both civil and military practice and is well adapted to the needs of the general practitioner at times called to operate with little time for preparation.

---

**Diseases of the Nose and Throat.** By Algernon Coolidge, M. D., Professor of Laryngology in the Harvard Medical School. 12mo of 360 pages, illustrated. Philadelphia and London: W. B. Saunders Company, 1915. Cloth, $1.50 net.

This is an admirable book of moderate pretensions to completeness and, indeed, containing more real material than many much larger works. The chapter on the tonsillar ring is especially valuable, giving a conception of the anatomic features and practical problems of this area which will be new to many.

---

**What to Eat and Why.** By G. Carroll Smith, M. D., of Boston, Mass. Second edition, thoroughly revised. Octavo of 377 pages. Philadelphia and London: W. B. Saunders Company, 1915. Cloth, $2.50 net.

Aside from brief tables, mostly in connection with certain metabolic diseases and a few receipts (sic) for foods designed especially for invalids, this work is confined to the therapeutics rather than the materia medica of diet. That is to say, diseases especially influenced by diet are considered seriatim, and the indications and contraindications are given for each, in a thorough manner.

---

**A Text-Book of Pathology.** By Alfred Stengel, M. D., Professor of Medicine, University of Pennsylvania, and Herbert Fox, M. D., Director of the Pepper Laboratory of Clinical Medicine, University of Pennsylvania. Sixth edition, reset. Octavo of 1045 pages, with 468 text-illustrations, many in colors, and 15 colored plates. Philadelphia and London: W. B. Saunders Company, 1915. Cloth, $6.00 net, half morocco, $7.50 net.

One of the pleasant features of the department of Reviews is that it frequently recalls personal associations. Dr. Stengel was a classmate of the writer and his pathology in an earlier edition was and is a vade mecum of practice. But we can speak impartially of the high quality of his book. We have previously mentioned that pathology, as a department of medicine, has been somewhat handicapped by the fact that it has been largely the work of young men who, with developing maturity of judgement and accumulated experience have used it as a stepping stone to medical or surgical practice. Very few have remained patholgists throughout their life, and it has happened that, of these few, still fewer have survived to old age. This fact, however, has its compensation in that pathology has been subjected to a practical point of view which has greatly influenced its adaptation by practitioners with no pretentions to expert pathologic skill. In the present instance, by the association with the original author of an expert, professional pathologist, there has been assured both the revision necessitated by the advance of the science of pathology and the practical, clinical experience and judgement needed to render the work adaptable to problems in medical practice.

---

**Principles and Practice of Obstetrics.** By Joseph B. DeLee, A. M., M. D. Professor of Obstetrics at the Northwestern University Medical School. Second edition, thoroughly revised. Large octavo of 1087 pages, with 938 illustrations, 175 of them in colors. Philadelphia and London: W. B. Saunders Company, 1915. Cloth, $8.00 net; half morocco, $9.50 net.

This is a massive work, covering in the most thorough manner the whole subject. The illustrations include not only the expected visualization of obstetric manoeuvres and pathology, but various kinds of records, filled in so as to possess a clinical value, a reproduction of the Semelweis monument and many other points of general interest. So far as medical science and practice can be reduced to print and picture, this work seems to us to be as nearly perfect as is humanly possible.

---

**How to Live.** By Irving Fisher, Ph. D., Chairman, Hygiene Reference Board of the Life Extension Institute, Inc., Professor of Political Economy, Yale University. 12mo, Cloth, 345 pages, indexed, illustrated. Price, $1.00, net; by mail, $1.12. Funk & Wagnalls Company, Publishers, New York.

**The Health-Cure of the Growing Child.** By Louis Fischer, M. D., Author of "Health-Care of the Baby," etc. Attending Physician in charge of the Babies' Ward of Sydenham Hospital, and to the Willard Parker and Riverside Hospitals; Former Instructor in Diseases of Children at the New York Post-Graduate Medical School and Hospital, etc. 12mo, Cloth, 354 pages, indexed, illustrated. Price, $1.25, net; by mail. $1.37. Funk & Wagnalls Company, Publishers, New York.

These two books may conveniently be reviewed together, though they are independent in authorship and the former is prepared in collaboration with the Hygiene Reference Board of the Life Extension Institute. Both are written by men competent to discuss the subject matter from a technical standpoint. Instead they have chosen to prepare books for the guidance of the laity and have succeeded, not only in reducing tchnicalities to popular understanding, but in avoiding the errors of statement and the probable errors of conception which most popular health books include. They have also succeeded as few technical students do, in presenting truths in a readable, popular style, without sacrifice of literary dignity.

**Orthopaedic Surgery.** Drs. Edward H. Bradford and Robert W. Lovett, Boston. Published by Wm. Wood & Co., New York. 5th edition, 416 pages, 368 illustrations, $3.25.

More than a quarter of this book is devoted to tuberculous lesions. The various conditions properly considered under orthopaedics are logically treated, with black letter headings for pathology, diagnosis, etc. The liberal use of illustrations elucidates the text, already clear. The work is of the highest order.

**Text Book of Nervous Diseases.** Charles L. Dana, A. M., M. D., LL.D., New York. Published by Wm. Wood & Co., New York. 8th edition. 632 pages, 262 illustrations. $4.25.

An interesting review of the scope of neurology is given in the preface to the first edition, no new diseases being listed in the preface to the present edition.

| | Peripheral | Spinal Cord | Brain | Functional | Total |
|---|---|---|---|---|---|
| Common and important | 31 | 13 | 12 | 10 | 66 |
| Rare | 56 | 27 | 16 | 11 | 110 |
| Totals | 87 | 40 | 28 | 21 | 176 |

The "and important" placed after common diseases is itself worth thinking about. Many authors implicitly put the "and important" after rare diseases. At the very beginning, we thus have an indication of the author's wholesome mental attitude. Over 10% of the volume is devoted to general considerations of anatomy, physiology, chemistry, hygiene, diagnosis and a critique of therapeutic measures. This broad preliminary treatment of the subject prepares the student, under- or post-graduate, for the seriatim consideration of special diseases which follows.

**Physiologic Chemistry.** Albert P. Mathews, Ph. D., Chicago. Published by Wm. Wood & Co., New York. 1040 pages, illustrated, $4.25.

While, in general, this book follows the standard arrangement, certain points are worth noting. It is replete with tables affording useful information on a great variety of topics. Some of the sub-headings are suggestive, as "Blood—The Circulating Tissue," "The Master Tissue of the Body," meaning that of the nervous system, "The Cryptorhetic Tissues," obviously referring to the internal secretions, though we think another r is needed. The only unfavorable criticism that we can offer is that the subject of caloric energy deserves a more extended and unified consideration, especially with regard to diet, and in comparison with various non-physiologic caloric problems. Part III, Practical Work and Methods, describes the equipment of a laboratory and many tests that can well be made by those interested in physiologic chemistry from the clinical standpoint. This book is more than a perfunctory treatise. It has the merit, seldom encountered, of giving more information than could be expected, along various lines that are capable of practical utilization.

---

**Diseases of Infants and Children.** Henry Dwight Chapin, A. M., M. D., and Godfrey Roger Pisek, M. D., Sc. D., New York. Published by Wm. Wood & Co., New York. 3d edition, 578 pages, 179 cuts and 12 colored plates. $3.25.

This work has already established itself as standard, in its earlier editions. It is divided into sections relating to the Newly Born, Hygiene of Infancy, Examination of the Sick Child, Infant Feeding, Digestive System (Apparatus please), Infectious Diseases, Respiratory Tract, etc., including the Commoner Surgical Diseases and the consideration of Specialties The illustrations add greatly to the descriptive power of the book. The work is extensive but not prolix, and is thoroughly practical in the broad sense of establishing practicality on a basis of scientific knowledge.

---

**The Model T Ford Car.** Victor W. Page, M. E. Published by the Norman W. Hanley Publishing Co., 132 Nassau Street, New York. 288 pages, 94 illustrations, $1.00.

This is a thoroughly practical book, not only for the Ford owner who expects to make his own repairs, but for the one who wishes a general understanding of the principles involved and a basis of judgement of the work of mechanics. It enters into greater detail than guides furnished with cars, and is not hampered by the consistent optimism of the salesman, to the effect that nothing will happen. It also possses a considerable educational value.

---

**The Practitioner's Visiting List for 1916.** Four styles: weekly, monthly, perpetual, sixty-patient. Pocket size; substantially bound in leather with flap, pocket, etc.; $1.25, net. Lea & Febiger, Publishers, Philadelphia and New York.

This Visiting List has proved its convenience by general use for many years. It needs no introduction except to those beginning practice.

---

**The Clinics of John B. Murphy, M. D.,** at Mercy Hospital, Chicago. Volume IV., Number V. (October 1915). Octavo of 228 pages, 56 illustrations. Philadelphia and London: W. B. Saunders Company, 1915. Published Bi-Monthly. Price per year: Paper, $8.00, Cloth, $12.00.

The October copy of Murphy's Clinics contains besides the usual case histories of bone work, several discussions of interest, such as two carcinoma cases with excision of the submaxillary lymph-nodes, two interesting cases of pain in amputation stumps, a good discussion of a case of pyonephrosis. etc.

